# Passive sensing at scale to advance the understanding of mental health

**DOI:** 10.1016/j.landig.2025.01.002

**Published:** 2025-03-01

**Authors:** Aiden Doherty, Sandra Bucci, Alexandra Kenny, Roman Kotov, Gosia Lipinska, Laura Ospina-Pinillos, Katharina Schultebraucks

**Affiliations:** 1 Nuffield Department of Population Health, University of Oxford; 2 Division of Psychology and Mental Health, School of Health Sciences, Faculty of Biology, Medicine and Health, Manchester Academic Health Sciences, The University of Manchester; 3 McPin Foundation, London, United Kingdom; 4 Department of Psychiatry, Stony Brook Medicine, Stony Brook, NY; 5 Department of Psychology, Humanities Faculty, University of Cape Town; 6 Department of Psychiatry and Mental Health, Pontificia Universidad Javeriana, Bogota, Colombia; 7 Department of Psychiatry, New York University Grossman School of Medicine, New York, NY, USA

## Introduction

Passive sensing devices such as smartphones and wearables provide a major opportunity to transform our understanding of the mechanisms, determinants, and consequences of mental health problems, including anxiety, depression, and psychosis. Most people in high- and low-income societies now own and regularly use passive sensing devices, where almost 70% of the world’s total population now uses a mobile device^1^. For example, despite limited resources, over 70% of the South African population use a smartphone^2^, while one-fifth of US adults own wearable technologies, like smartwatches and fitness trackers.^1^ These devices typically have a range of embedded sensors, such as accelerometers, that offer the potential to continuously, noninvasively, and painlessly measure traits that are relevant to mental health such as physical activity, sleep, and circadian rhythms. If harnessed effectively, this high level of health-relevant data would advance understanding and early intervention for mental health problems and lower the barriers for researchers and clinicians to start or continue using digital technologies for mental health. However, given the complexity of mental health measurement, it is important to address some key challenges so that data from smartphones and wearables can reliably inform the future care of people living with mental health difficulties.

## Value Proposition

Smartphones and wearables offer the opportunity to improve how we understand, predict, prevent, detect, and treat mental health problems. For example, an important element of depression diagnosis and monitoring is to consider symptoms self-reported by patients. In contrast, passive sensing devices offer the potential to identify people who encounter changes in their activity levels, sleep pattern and daily movements just before they become unwell or experience a relapse. The wide range of data from these devices could provide a much clearer picture of the daily rhythms of health and well-being, as well as the environment in which these occur. The touch screens, motion sensors, microphones, cameras, location sensors, and other technologies within these devices allow us to rethink how we measure things that might be important and relevant to mental health research. For risk factors such as physical activity, wrist-worn devices offer an opportunity to shift from the use of subjective questionnaires to the continuous objective measurement of physical activity patterns. The use of passive sensors could clearly improve the granularity, validity, reliability, and collection of some data relevant to mental health.

## Problem Statement

However, the current evidence base is not sufficiently mature to support the use of symptoms measured from smartphones and wearables to reliably inform the care of people living with mental health problems. Flagship studies such as AURORA^2^ or CONNECT^3^ will provide valuable new evidence in clinical populations. Nevertheless, to mitigate against bias arising from underpowered case-control studies, smartphones and wearables have not been used for mental health research at the scale of tens or hundreds of thousands of participants in prospective longitudinal studies in populations with a high burden of mental health difficulties. Although the UK Biobank is one of the most important medical resources worldwide^3^ and contains a wearable assessment of physical activity and sleep in 100,000 participants^4^; the study population (median age at baseline of 62 years, IQR 54–68 years) is not ideal to examine the causes of mental health problems. This is because it can only look at mental health in the older population, where depression and Alzheimer’s can occur, and it is important to note that 75% of mental health problems start before the age of 24 years^5^.

### Beyond study design issues, there also exists a lack of agreed technical standards and associated training on how to measure relevant traits from passive sensor data

For example, in the field of physical activity, there are numerous proposed methods^6^, sensors, metrics, and little consensus on how to record a simple measurement such as daily step count. It is also recognised that analytic decisions such as the temporal resolution selected for summarising and analysing sensor data will affect how results are interpreted^7^. In addition, it is difficult to maintain research analysis pipelines due to frequent software updates from manufacturers of consumer wearables. Furthermore, passive sensors such as optical photoplethysmogram sensors might introduce bias where light is absorbed differently, and hence the algorithm output might be less accurate, in patients with darker skin tones versus those with light skin tones. Research capacity to address these important issues is currently limited due to a lack of relevant health data science and technical skills within mental health study teams.

Finally, ethical and privacy challenges exist for passive sensors such as Bluetooth and global positioning sensors that are good to infer social connectiveness and mobility respectively but in both cases might reveal unwanted data on who someone met or where they went. It is important to address these major challenges for smartphones and wearables to move beyond promise and towards delivery of new insights and more responsive care for the wider mental health community.

## Recommendations For Passive Sensing In Mental Health Research

In September 2023, Wellcome and Google partnered to host a convening of a diverse set of clinicians, biomedical and computer scientists, funding agencies, and lived experience experts to identify recommendations to advance the use of passive sensing in mental health research.

### Establish a biobank of passive sensors in populations with the highest burden of mental health problems

Studies such as the UK Biobank, All of Us^8^, and China Kadoorie Biobank^9^, provide the template and impetus for a resource of a similar scale in populations at high risk of mental health difficulties (such as adolescents and younger adults) to help transform our understanding of the causes and consequences, prevention, and treatment of mental health problems. These studies have included passive sensing measurements in tens and hundreds of thousands of participants, with linkage to subsequent diagnoses of ill health. Furthermore, they offer a mechanism to promote innovative science by maximising global access to the data in an equitable and transparent manner. A new study would need careful consideration of target populations of interest, which should be determined in consultation with multiple stakeholders globally. An important requirement will be linkage to clinical patient notes, and validated questionnaires (that could be collected via a smartphone platform). The establishment of this biobank, or enhancement of relevant existing biobanks, would revolutionise our ability to evaluate the effectiveness of passive sensing data in prognosticating mental health risk and inform future strategies to develop new interventions.

### Build large-scale validation datasets to support an industry-academia consortium around the analysis of passive sensor data

Ready access to such a large-scale biobank for mental health research, with passive sensor measurements, would coalesce researchers from many disciplines across academia and industry to collaborate to ensure that different types of complex passive sensor data are appropriately analysed. To this end, collaboration between researchers and industry, to achieve cross-platform metrics for previously identified variables of interest, such as sleep quantity, quality and regularity (e.g. point of sleep onset, wake after sleep onset) and physical activity (step count, heart rate) would allow for reliable interpretation of large data sets that are pooled from multiple studies, to infer robust prediction, detection and treatment of mental health conditions. To achieve this, it is important to design a range of supporting validation datasets with reference measurements such as polysomnography for sleep, or camera footage of steps for physical activity. In addition, the creation of new public-private partnerships could provide a forum to provide advance notice of software updates, which have been disruptive, or fatal, to past research projects. If a series of collaborative validation studies are successfully implemented, this initiative would provide much more clarity to researchers and clinicians around how to preprocess, analyse, and interpret multiple modalities of passive sensing data.

### Design ecosystems to encourage ethical use of passive sensors in health research

Passive sensors such as GPS are likely to offer important new measurements but also have associated privacy trade-offs. It is therefore important to bring together humanities researchers to lead the debate on the ethical requirements for scientific research and technological innovation, capable of commanding well-founded public trust and confidence. Such a process will need to be co-designed and co-developed with Lived Experience experts from different cultures and locations to ensure equity. This includes addressing inequities in accessing mental health support and digital technology (e.g., in lower resource settings, not settling for “poor tech for poor people”). The need to include groups currently under-represented in mental health research remains a key consideration. Technology navigators will be important in helping some groups of patients move through the complex care continuum and technology, with the goal of facilitating information flow and resource accessibility for research and clinical outcomes. In addition, the input of Lived Experience, and other relevant, experts will be important to co-create safe and effective data access procedures to any new research studies created. This would help identify solutions to ensure safe data access to approved researchers and clinicians, somewhat similar to procedures used by the CVD-COVID-UK consortium to provide researcher access to relevant NHS England data^10^.

## Conclusions

Smartphones and wearables offer the opportunity to improve how we predict, prevent, detect, and treat mental health problems. However, large scale biobanks containing these measurements in a standardized way in populations with high burdens of mental health difficulties are needed. Supported by diverse validation datasets to address device heterogeneity and an ecosystem of ethical guidance, we can then reliably determine if passive sensing can inform the future care of people living with mental health problems.

## Footnotes

1
https://datareportal.com/reports/digital-2024-global-overview-report

2
www.statista.com/statistics/488376/forecast-of-smartphone-users-in-south-africa/

3
https://www.connectdigitalstudy.com/
